# GC-072, a Novel Therapeutic Candidate for Oral Treatment of Melioidosis and Infections Caused by Select Biothreat Pathogens

**DOI:** 10.1128/AAC.00834-19

**Published:** 2019-11-21

**Authors:** Jeffry D. Shearer, Michelle L. Saylor, Christine M. Butler, Anthony M. Treston, Henry S. Heine, Sunisa Chirakul, Herbert P. Schweizer, Arnold Louie, George L. Drusano, Steven D. Zumbrun, Kelly L. Warfield

**Affiliations:** a Emergent BioSolutions, Gaithersburg, Maryland, USA; b Institute for Therapeutic Innovation, University of Florida, College of Medicine, Orlando, Florida, USA; c Emerging Pathogens Institute, University of Florida, College of Medicine, Gainesville, Florida, USA; d United States Army Medical Research Institute of Infectious Diseases, Frederick, Maryland, USA

**Keywords:** melioidosis, *Burkholderia*, antibiotic, GC-072, biodefense

## Abstract

Burkholderia pseudomallei, the etiological agent of melioidosis, is a Gram-negative bacterium with additional concern as a biothreat pathogen. The mortality rate from B. pseudomallei varies depending on the type of infection and extent of available health care; in the case of septicemia, left untreated, it can range from 50% to 90%. Current therapy for melioidosis is biphasic, consisting of parenteral acute-phase treatment for 2 weeks or longer, followed by oral eradication-phase treatment lasting several months. An effective oral therapeutic for outpatient treatment of acute-phase melioidosis is needed. GC-072 is a potent, 4-oxoquinolizine antibiotic with selective inhibitory activity against bacterial topoisomerases. GC-072 has demonstrated *in vitro* potency against susceptible and drug-resistant strains of B. pseudomallei and is also active against Burkholderia mallei, Bacillus anthracis, Yersinia pestis, and Francisella tularensis. GC-072 is bactericidal both extra- and intracellularly, with rapid killing noted within a few hours and reduced development of resistance compared to that for ceftazidime. GC-072, delivered intragastrically to mimic oral administration, promoted dose-dependent survival in mice using lethal inhalational models of B. pseudomallei infection following exposure to a 24- or 339-LD_50_ (50% lethal dose) challenge with B. pseudomallei strain 1026b. Overall, GC-072 appears to be a strong candidate for first-line oral treatment of melioidosis.

## Abstract (precis)

Burkholderia pseudomallei, the etiological agent of melioidosis, is a Gram-negative bacterium with additional concern as a biothreat pathogen. The mortality rate from B. pseudomallei varies depending on the type of infection and extent of available health care; in the case of septicemia, left untreated, it can range from 50% to 90%. Current therapy for melioidosis is biphasic, consisting of parenteral acute-phase treatment for 2 weeks or longer, followed by oral eradication-phase treatment lasting several months.

## INTRODUCTION

Burkholderia pseudomallei is a Gram-negative bacterium which is the etiological agent of melioidosis, a serious and sometimes fatal disease (1, 2). B. pseudomallei is endemic to Southeast Asia and northern Australia, where melioidosis is a particularly serious health problem associated with a mortality rate of approximately 50% in northeast Thailand and approximately 20% in Australia (2–5). The geographic distribution of B. pseudomallei is likely substantially larger than previously thought, encompassing many other tropical and subtropical regions where the disease has yet to be reported. In the countries where melioidosis is known to be endemic, it is suspected that the burden of disease is higher than estimated due to underdiagnosis and underreporting (1, 2, 4–6).

B. pseudomallei can be found in soil and water, and infection typically occurs after environmental exposure during occupational or recreational activities, often following the rainy season and severe weather events. Most commonly, infections are acquired via percutaneous inoculation, inhalation of bacteria, or ingestion of a contaminated water supply (5, 7–9). Infection following inhalation of B. pseudomallei often leads to more severe pneumonic illness (3, 10–12). Due to its infectivity via inhalation or contact with skin, as well as its moderate ease of dissemination and mortality rates, B. pseudomallei is a pathogen of interest for its potential to be weaponized and used as an agent of biowarfare. B. pseudomallei is classified as a category B biothreat agent by the National Institute of Allergy and Infectious Diseases (NIAID) (13, 14) and a tier 1 select agent by the Centers for Disease Control and Prevention (CDC) and United States Department of Agriculture (USDA) due to its potential to cause a severe threat to human health (15).

Melioidosis has a broad range of clinical presentations, including acute fulminant pneumonia, genitourinary infection, septicemia acquired by inhalation, and wound infections acquired by inoculation of bacteria from soil through abraded skin (1, 3, 6, 7, 16). Current therapy for melioidosis is prolonged and the risks of failure and relapse are high. Treatment is biphasic, requiring both intravenous and oral step-down treatment phases consisting of two or more weeks of parenteral acute-phase treatment with intravenous (i.v.) ceftazidime, followed by an eradication phase with oral amoxicillin-clavulanic acid or trimethoprim-sulfamethoxazole administered over several additional months (4, 17, 18). A major feature of B. pseudomallei is the ability to remain latent in the host, causing relapse infections years after the initial infection (8). Between 6% and 13% of melioidosis cases occurring within a year of primary infection are due to relapse rather than reinfection (19). Trauma or immunosuppression is associated with emergence of bacteria that were dormant for prolonged periods (20–22). B. pseudomallei is believed to have the ability to enter a dormant state in an intracellular location, where it can avoid immunological clearance (9).

Antibiotic resistance is currently a major global health concern, and there is an immediate unmet medical need to develop new effective treatments against resistant pathogens (23–25). While the incidence of melioidosis is relatively small in comparison to that of pathogens with more widespread antibiotic resistance, (e.g., Staphylococcus aureus), an unmet medical need for effective treatment of melioidosis still exists, as current treatment options are limited. Due to poor clinical performance, existing fluoroquinolones are currently not recommended for the treatment of melioidosis except in cases where resistance or intolerance to other available antibiotics is known (26).

B. pseudomallei exhibits resistance to diverse antibiotics, including first- and second-generation cephalosporins, penicillins, macrolides, and aminoglycosides (2, 3, 27–32). Ceftazidime- and clavulanic acid-resistant strains have been described, and others continue to be identified (26, 31, 33–37). These strains, in particular, contain mutations in the *penA* gene, resulting in a change in the amino acid sequence of PenA β-lactamase or its expression (33–39). Although PenA is the major acquired ceftazidime resistance mechanism, other ceftazidime resistance mechanisms exist, including deletion of penicillin-binding protein 3 (40). Efflux via pumps belonging to the resistance nodulation cell division family comprises the sole multidrug resistance mechanism documented thus far in B. pseudomallei (28, 41). Three efflux pumps have been characterized in some detail. AmrAB-OprA is expressed in most B. pseudomallei strains and has been implicated in intrinsic and acquired resistance to aminoglycosides and macrolides (27, 30, 37, 42–44). Although BpeAB-OprB is expressed at detectable levels in wild-type strains, its clinical significance remains unclear because of the modest levels of drug resistance it causes, even in strains with increased pump expression (29, 41). Efflux via BpeEF-OprC is the main fluoroquinolone resistance mechanism in B. pseudomallei; the BpeEF-OprC substrate spectrum also includes tetracyclines, chloramphenicol, and both components of the preferred eradication-phase therapy, trimethoprim and sulfamethoxazole (28, 30, 41, 45–49).

Bacterial type IIA topoisomerases are highly potent clinically validated targets for antimicrobial agents, as demonstrated by the clinical and commercial success of the fluoroquinolone class of topoisomerase inhibitors, such as ciprofloxacin, levofloxacin, and moxifloxacin (50). However, fluoroquinolones are not currently recommended for treating melioidosis because of a high incidence of therapeutic failures in clinical studies (17, 51). Bacterial resistance to quinolone compounds is predominantly due to target protein mutations, although efflux- and plasmid-mediated resistance mechanisms are also known (52, 53). The amino acid changes in DNA gyrase and topoisomerase IV (Topo IV) leading to fluoroquinolone resistance occur mainly in the quinolone resistance-determining region (QRDR) of respective subunits GyrA and ParC (54, 55).

**FIG 1:**
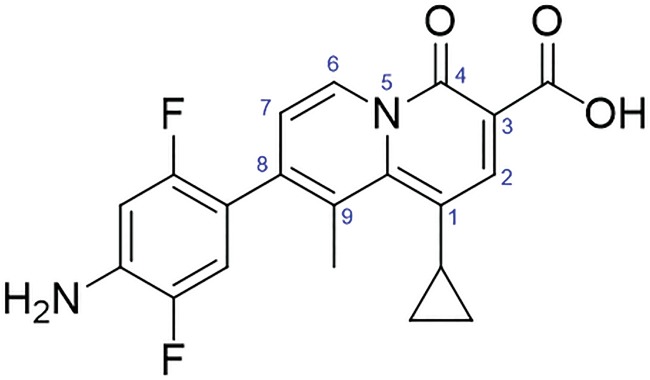
GC-072 is a novel topoisomerase inhibitor. The position of nitrogen in the heteroaromatic structure confers significantly different physicochemical and biological properties of GC-072 than those of fluoroquinolone-class antibiotics.

Modifying the structure of existing antibiotics to increase potency and overcome mechanisms associated with resistance provides a more practical approach than finding new antibacterial agents with novel mechanisms of action (56). GC-072 (Fig. 1) is a 4-oxoquinolizine, a class for which there were a number of publications and patents in the 1990s, mostly from Abbott Laboratories; however, relatively little about this class has been recently reported. There are no available literature reports indicating clinical development of 4-oxoquinolizines. The therapeutic targets of oxoquinolizines, such as GC-072, are the bacterial DNA gyrase and Topo IV enzymes, collectively known as type IIA topoisomerases. Oxoquinolizines, which include GC-072, possess potent antimicrobial activity against a broad spectrum of organisms, including Gram-positive, Gram-negative, and resistant bacteria, including those with mutations in the QRDR that confer quinolone resistance (57–60). The position of nitrogen in the heteroaromatic structure of GC-072 confers different physicochemical and biological properties than those of the fluoroquinolone class of antibiotics. The C-8 group has been demonstrated to have importance in terms of functionality for oxoquinolizines (also known as 2-pyridones) (57, 60).

The results presented here from *in vivo* studies indicate GC-072 is a promising candidate for the oral treatment of acute B. pseudomallei-associated pneumonic infection. Current first-line standard-of-care treatment for melioidosis is i.v. ceftazidime (17), which does not have an oral option. The replacement of acute-phase i.v. treatment with an effective oral alternate would greatly enhance health care options for melioidosis treatment, particularly in resource-constrained settings or in the event of an intentional release (biowarfare).

## RESULTS

### *In vitro* selectivity of GC-072 for bacterial and human topoisomerases.

GC-072 was tested in comparison to ciprofloxacin using gel-based topoisomerase assays (Table 1). Results of these assays are reported as the half-maximal inhibitory concentration (IC_50_) for inhibiting activity of bacterial topoisomerases II (DNA gyrase) and IV and human topoisomerases I and II (Topo I and Topo II, respectively). GC-072 inhibited both Escherichia coli and S. aureus gyrase and Topo IV at concentrations comparable to, or lower than, those for ciprofloxacin. GC-072 more actively inhibited DNA gyrase isolated from quinolone-resistant E. coli than ciprofloxacin, implying GC-072 has a different binding mode than fluoroquinolones such as ciprofloxacin. Furthermore, GC-072 demonstrated no detectable inhibition of human Topo I and II, comparable to that of ciprofloxacin, indicating that it is selective for bacterial topoisomerases and likely to have an acceptable genotoxicity profile.

**TABLE 1:** *In vitro* selectivity of GC-072 for bacterial and human topoisomerases

Compound	IC_50_ (μM)						
	Bacterial topoisomerases					Human topoisomerases	
	S. aureus		E. coli				
	Gyrase	Topo IV	Gyrase	Quinolone-resistant gyrase	Topo IV	Topo I	Topo II
GC-072	2	4–30	0.18–1.50	1.30–1.50	4.22–8.45	>100	>100
Ciprofloxacin	62	15–30	0.16–1.68	35–130	2.35–4.71	>100	>100

### *In vitro* susceptibility of B. pseudomallei to GC-072.

Susceptibility testing was performed to determine the MICs of GC-072 for 100 strains of B. pseudomallei. These strains represent the geographic distribution across Southeast Asia. The majority are clinical isolates and include strains resistant against “front-line” antibiotic therapies, including ceftazidime, carbapenem, and tetracycline. GC-072 demonstrated good activity against B. pseudomallei, with an MIC_90_ of 0.25 μg/ml and a range of ≤0.008 to 1 μg/ml (Table 2). This compares to MIC_90_s of 8, 8, 1, 64, and 4 μg/ml for ciprofloxacin, finafloxacin, meropenem, ceftazidime, and doxycycline, respectively. The quality control strain MIC results were all within CLSI ranges for the comparator antibiotics.

**TABLE 2:** *In vitro* susceptibility of B. pseudomallei to GC-072 and comparators

Antibiotic	No. of strains	MIC (μg/ml)^*a*^		
		Range	50%	90%
GC-072	100	≤0.008 to 1	0.12	0.25
Ciprofloxacin	100	0.25 to 16	2	8
Finafloxacin	50	1 to ≥16	4	8
Meropenem	100	≤0.06 to 4	0.5	1
Ceftazidime	100	0.25 to ≥128	1	64
Doxycycline	100	≤0.06 to 32	0.5	4

a Antimicrobial activity was determined by the microdilution method in 96-well plates according to CLSI guidelines. All compounds were tested in parallel against the recommended CLSI reference quality control strains E. coli (ATCC 25922) and P. aeruginosa (ATCC 27853).

### *In vitro* susceptibility of additional bacterial biothreat pathogens to GC-072.

Susceptibility testing was performed to determine the activity of GC-072 and antibiotic comparators against four additional biothreat agents. GC-072 was tested against 30 geographically biodiverse strains each of category A biothreat agents Bacillus anthracis, Yersinia pestis, and Francisella tularensis and category B biothreat agent Burkholderia mallei. Overall, GC-072 exhibited strong *in vitro* antimicrobial potency against all four biothreat pathogens. The results are summarized in Table 3. The MIC_90_s of GC-072 against B. anthracis, Y. pestis, F. tularensis, and B. mallei were 0.002, 0.015, ≤0.0005, and 0.12 μg/ml, respectively. GC-072 demonstrated increased activity compared to those of all antibiotic comparators, including the fluoroquinolone finafloxacin, with lower MIC_90_ values for all pathogens tested. The quality control strain MIC results were all within CLSI ranges for the comparator antibiotics.

**TABLE 3:** *In vitro* antibiotic susceptibility of additional biodefense pathogens

Pathogen	Antibiotic	MIC (μg/ml)^*a*^		
		Range	50%	90%
B. anthracis (*n* = 30)	GC-072	≤0.0005–0.004	0.001	0.002
	Finafloxacin	0.03–0.12	0.06	0.12
	Ciprofloxacin	0.015–0.06	0.03	0.06
F. tularensis (*n* = 30)	GC-072	≤0.0005–0.015	≤0.0005	≤0.0005
	Finafloxacin	≤0.004–0.12	≤0.004	0.008
	Ciprofloxacin	≤0.004–1	0.008	0.015
Y. pestis (*n* = 30)	GC-072	0.002–0.03	0.008	0.015
	Finafloxacin	0.015–0.25	0.06	0.12
	Ciprofloxacin	≤0.004–0.12	0.015	0.03
B. mallei (*n* = 30)	GC-072	≤0.004–0.5	0.015	0.12
	Finafloxacin	0.015–2	0.25	2
	Azithromycin	0.12–1	0.5	0.5

a MICs were determined by the microdilution method in 96-well plates according to CLSI guidelines. All compounds were tested in parallel against the recommended CLSI reference quality control strains E. coli ATCC 25922, S. aureus ATCC 29213, and P. aeruginosa ATCC 27853.

### Mutation frequency.

The mutation frequencies to 3× the MICs of GC-072 and ceftazidime for B. pseudomallei 1026b were determined in a total of 11 experiments (6 with GC-072 and 5 with ceftazidime in parallel). The mutation frequency for GC-072 ranged from −7.1 log to −8.4 log CFU. The MICs of a subset of colonies that grew on GC-072-supplemented agar ranged from 0.5 to 2 mg/liter compared with an MIC of 0.125 to 0.25 mg/liter for the parent strain. The mutation frequency values for ceftazidime were −6.6 log to −8.0 log CFU. The colonies that grew on the ceftazidime-supplemented agar plates had MICs of 4 to 32 mg/liter, compared to an MIC of 1 to 2 mg/liter for the parent strain.

### Time-kill study.

**FIG 2:**
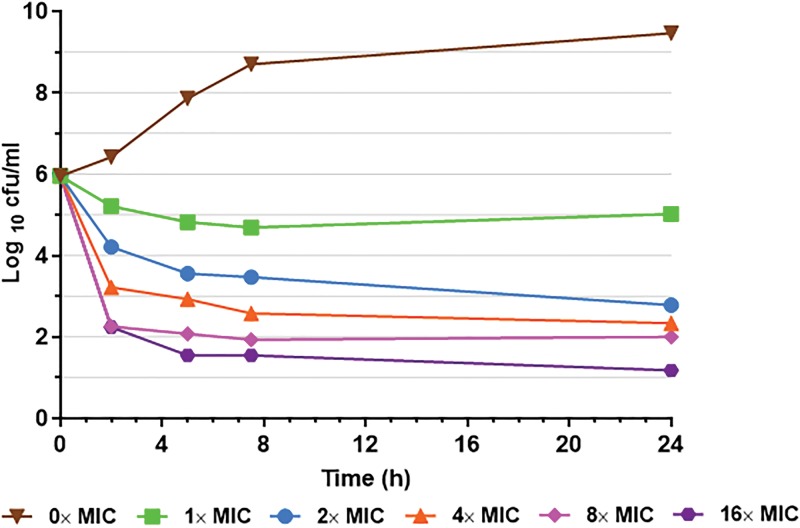
*In vitro* time-kill assay. Concentration-dependent activity of GC-072 against B. pseudomallei strain 1026b over a 24-h period. Bacterial suspensions containing B. pseudomallei strain 1026b were left untreated or inoculated with GC-072 at concentrations of 1×, 2×, 4×, 8×, or 16× MIC (0.125 mg/liter). At 0, 2, 5, 7.5, and 24 h of incubation, samples were collected from each suspension, quantitatively cultured, and enumerated after 48 h of incubation.

An *in vitro* time-kill assay was performed to measure the rate and activity of GC-072 in killing B. pseudomallei at different concentrations. Bacterial suspensions containing B. pseudomallei strain 1026b were left untreated or inoculated with GC-072 at concentrations of 1×, 2×, 4×, 8×, or 16× MIC and incubated over a 24-h period. Samples were collected from each suspension at five time points and enumerated. Untreated bacteria in the control group grew to titers >1 × 10^9^ CFU/ml, with maximal growth noted between 8 and 24 h (Fig. 2). GC-072 provided a dose-response effect on the reduction of viable B. pseudomallei 1026b. GC-072 demonstrated rapid bactericidal activity at all concentrations. As shown in Fig. 2, more than 75% of the full effect was observed within 5 h. Regrowth was not seen in any of the treatment arms, indicating that the killing effect of GC-072 was sustained. While complete killing was not observed at any of the GC-072 concentrations tested, at 24 h, the 8× and 16× MIC bacterial counts were at, or near, the lower limit of quantitation (100 and 15 CFU/ml, respectively). The limits of solubility of GC-072 in aqueous media may have resulted in reduced incremental killing between the 8× and 16× concentrations compared with the increments in killing between the lower concentrations. Alternatively, slow precipitation of the drug out of solution over time may have contributed to the lack of additional killing past the 8-h time point and, hence, the lack of sterilization of the cultures. Bacterial samples of GC-072-supplemented agar were not plated after treatment; therefore, it is unknown if resistant subpopulations may have emerged as a result of drug exposure in this particular study.

### GC-072 is active against drug-resistant B. pseudomallei.

Two studies were conducted to investigate the activity of GC-072 against B. pseudomallei strains with characterized antibiotic resistance mechanisms. An isogenic panel of B. pseudomallei 1026b-based efflux-proficient and -compromised strains that either expressed or lacked defined efflux pumps were used to assess the efflux propensity of GC-072. Doxycycline and ciprofloxacin were used as control antibiotics, as they are prone to efflux, particularly by the BpeEF-OprC pump in B. pseudomallei (41, 48). The observed MIC for GC-072 against B. pseudomallei strain 1026b was within the previously observed MIC range for diverse B. pseudomallei strains, including other studies with 1026b (Table 4). As demonstrated by an MIC comparable to the parental 1026b strain, GC-072 does not appear to be effluxed by strains solely expressing AmrAB-OprA (e.g., Bp227), and efflux by BpeAB-OprB is barely discernible (e.g., Bp58). GC-072 appears to be a substrate of BpeEF-OprC, as indicated by the increased MIC in strain Bp282 compared to that of strains either not expressing (e.g., 1026b) or lacking (e.g., Bp320) the BpeEF-OprC efflux system. However, the degree of efflux of GC-072 by BpEF-OprC was significantly lower than that observed with comparators ciprofloxacin and doxycycline, and the observed susceptibility (MIC, 0.5 to 1 μg/ml) is within the range reported above for 100 clinical isolates.

**TABLE 4:** Activity of GC-072 and comparators against efflux-proficient strains of B. pseudomallei

Strain^*a*^	Genotype or description	Efflux pump(s) expressed	MIC (μg/ml)^*b*^		
			Doxycycline	Ciprofloxacin	GC-072
1026b	Wild type	AmrAB-OprA, BpeAB-OprB	0.5	2	0.25
Bp340	1026b Δ(*amrAB-oprA*)	BpeAB-OprB	0.125	0.5–1	0.063
Bp227	1026b Δ(*bpeAB-oprB*)	AmrAB-OprA	0.125	0.5	≤0.004–0.016
Bp207	1026b Δ(*amrAB-oprA*) Δ(*bpeAB-oprB*)	None known	≤0.008	0.25	≤0.004–0.031
Bp58	1026b Δ*bpeR* Δ(*amrAB-oprA*)	BpeAB-OprB	0.5	2	0.25
Bp282	Bp207 *bpeT*	BpeEF-OprC	4	16	1
Bp320	Bp282 Δ(*bpeEF-oprC*)	None known	≤0.0078125	0.25–0.5	≤0.004–0.125
ATCC 25922^*c*^	*E. coli* ATCC 25922		1	≤0.008	≤0.004
ATCC 27853^*c*^	*P. aeruginosa* ATCC 27853		>16	0.25	1

a All strains used in this study are derived from B. pseudomallei 1026b as previously described, with the exception of Bp282 and Bp320. The latter two strains were derived as follows: Bp282 is a ciprofloxacin-resistant derivative of Bp207 obtained using passive selection. It contains a *bpeT* point mutation causing an S280P BpeT amino acid substitution. The passive selection experiment was conducted prior to 4 December 2012, and its performance and mutant possession did not require US Federal Select Agent Program approval. Bp320 is a Δ(*bpeEF-oprC*) derivative of Δ(*amrAB-oprA*) Δ(*bpeAB-oprB*) strain Bp282 and was constructed using the previously described methods. This strain does not express any known efflux pumps (48).

b The MIC against each strain was determined using broth microdilution assays. The data shown represent three biological replicates, each in technical duplicates, reporting modes from 6 data points.

c The control strains E. coli ATCC 25922 and P. aeruginosa ATCC 27853 were verified in parallel with all MIC testing to ensure that MICs for the control antibiotics were within the expected range, according to CLSI guidelines.

In a separate study, a panel of select agent-excluded B. pseudomallei strain Bp82 derivatives was employed to assess single drug class resistance due to mutations causing β-lactam resistance (e.g., ceftazidime resistance due to PenA β-lactamase point mutations or overexpression or trimethoprim resistance due to *folA* mutation). The results show that GC-072 was fully active against the panel of drug-resistant B. pseudomallei strains tested (i.e., ceftazidime-, clavulanate- and trimethoprim-resistant strains) and its activity was superior to that of ciprofloxacin in this panel (Table 5).

**TABLE 5:** Activity of GC-072 against drug-resistant strains of B. pseudomallei

Strain^*a*^	Phenotype	MIC (μg/ml)^*b*^		
		Ceftazidime	Ciprofloxacin	GC-072
Bp82 (*penA^+^*)	Wild type	2	2	0.5
Bp82 Δ*penA*	PenA β-lactamase deficient	0.5	2	0.5
Bp82 *penA*_C69Y_	Ceftazidime resistant	64	2–4	0.5
Bp82 *penA*_P167S_	Ceftazidime resistant	8	4	0.5
Bp82 *penA*_D240G_	Ceftazidime resistant	8–16	2–4	0.25–1
Bp82 *penA*_S72F_	Clavulanate resistant	1	2–4	0.5
Bp82 P*_tac_-penA*^+^	PenA β-lactamase overproducer	16	2	0.5
Bp82 *folA*_I99L_	Trimethoprim resistant	1–2	4	0.5
*E. coli* ATCC 25922^*c*^	Wild type	0.25	≤0.016	0.008
*P. aeruginosa* ATCC 27853^*c*^	Wild type	4	0.25	1

a A panel derived from the attenuated strain Bp82 (47) was used to assess single drug class resistance due to target mutations causing β-lactam resistance (e.g., ceftazidime resistance due to PenA β-lactamase point mutations or overexpression) or trimethoprim resistance (due to *folA* point mutations).

b The MIC against each strain was determined using broth microdilution assays. The data shown represent three biological replicates, each in duplicates, reporting modes from 6 data points.

c Values for ceftazidime and ciprofloxacin obtained with E. coli and P. aeruginosa type strains are within the expected CLSI range.

### GC-072 is active in a model of B. pseudomallei intracellular survival.

**FIG 3:**
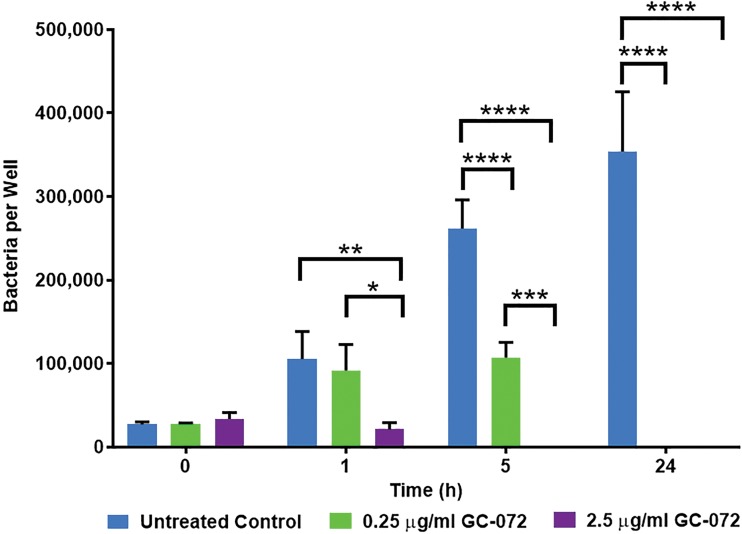
Intracellular activity. The activity of GC-072 against intracellular B. pseudomallei 1026b was assessed via intracellular survival assays. Murine RAW 264.7 macrophages were infected with B. pseudomallei strain 1026b at an MOI of ∼10. Cells were either left untreated or treated with 0.25 mg/ml or 2.5 mg/ml of GC-072. Residual bacteria counts were assessed from lysed cells at 0, 1, 5, and 24 h after initiation of treatment. Error bars indicate the errors of the means from three biological replicates. *, *P* ≤ 0.05; **, *P* < 0.01; ***, *P* < 0.001; ****, *P* < 0.0001.

The activity of GC-072 against intracellular B. pseudomallei 1026b was assessed via intracellular survival assays. The MIC for B. pseudomallei strain 1026b measured for this experiment was 0.25 μg/ml and was used to set the multiples of the MICs for the intracellular survival assay. Cells were either left untreated or treated with 0.25 μg/ml or 2.5 μg/ml of GC-072. The results from this study indicate GC-072 is effective at inhibiting growth of intracellular B. pseudomallei in murine macrophage cells *in vitro*. As shown in Fig. 3, at dose levels close to the MIC (0.25 μg/ml) or approximately 10× the MIC (2.5 μg/ml), GC-072 effectively inhibited the growth of intracellular B. pseudomallei in a rapid time- and dose-dependent manner. After 1 h, the numbers of bacteria were significantly reduced in cultures treated with the higher GC-072 concentration compared to those in untreated cultures (*P* < 0.0023) (see Tables S1 and S2 in the supplemental material). At 5 h, there was a significant reduction in B. pseudomallei at both GC-072 concentrations compared to untreated B. pseudomallei, both having a *P* value of <0.0001 for cultures treated with 0.25 and 2.5 μg/ml. Twenty-four hours after initiation of treatment, there were no viable bacteria detectable at either the lower (0.25 μg/ml) or higher (2.5 μg/ml) concentrations of GC-072, compared to a mean of 3.5 × 10^5^ bacteria per well in the untreated culture (Tables S1 and S2). Ceftazidime is traditionally not employed as a comparator drug in intracellular survival models due to poor macrophage permeability; ceftazidime concentrations as high as 10 μg/ml have no effect on intracellular survival of B. pseudomallei in macrophages (61). Thus, ceftazidime was not used as a positive control in this study.

### GC-072 is efficacious against B. pseudomallei aerosol infection in a mouse model following exposure to a 24-LD_50_ bacterial challenge.

An *in vivo* murine melioidosis model of pneumonic illness was employed to determine the efficacy of GC-072 against an aerosol challenge with B. pseudomallei strain 1026b. BALB/c mice (*n* = 10 mice/group) were treated starting at 8 or 24 h postchallenge with either GC-072 (1, 3, 10, or 30 mg/kg) administered three times a day intragastrically (i.g.), 150 mg/kg of ceftazidime delivered intraperitoneally (i.p.) four times daily, or vehicle administered i.g. thrice daily. For the challenge, mice were divided into two exposure groups balanced such that each cohort received five animals from each of the two aerosol runs. The actual bacterial exposures were 6.37 × 10^4^ and 1.08 × 10^5^ CFU/mouse for each of the two groups. These represented 17.7 and 30 LD_50_s, respectively, with a mean of 23.8 LD_50_s.

**FIG 4:**
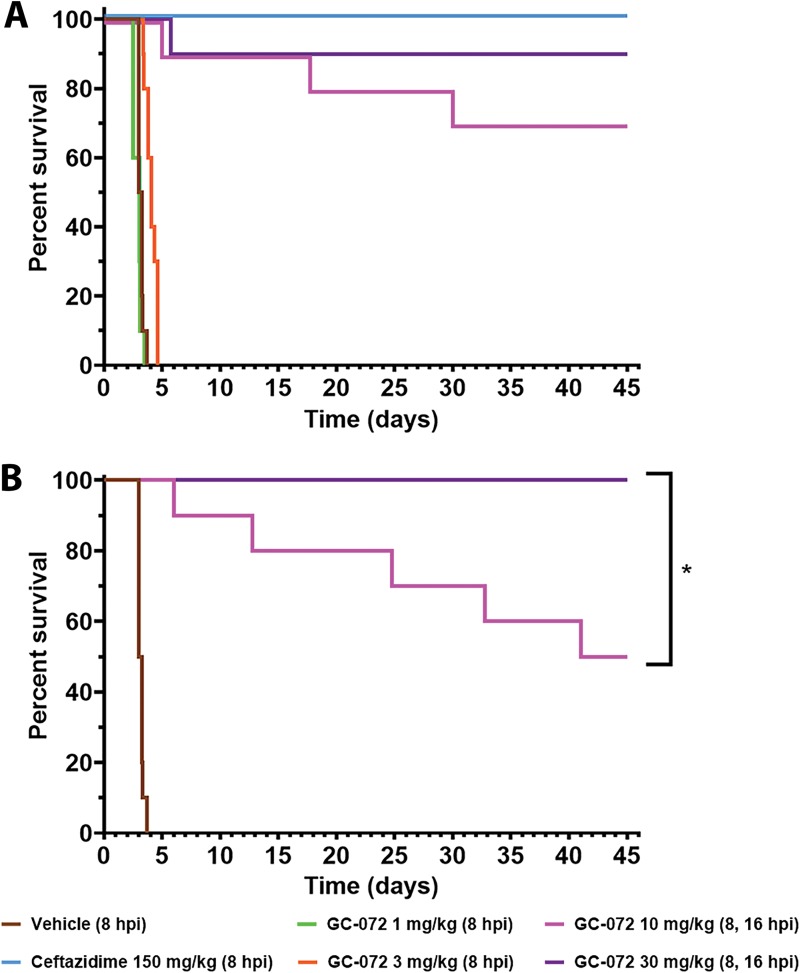
*In vivo* efficacy of GC-072 in a mouse melioidosis model following aerosol exposure to a 24-LD_50_ bacterial challenge with B. pseudomallei strain 1026b. Treatment initiation is indicated in parentheses. Following the challenge, female BALB/c mice (*n* = 10 per group except as noted below; age, 6 to 8 weeks) were treated i.g. via oral gavage with vehicle (SWFI), GC-072 (1, 3, 10, or 30 mg/kg, formulated as suspension with 0.5% methylcellulose) q8h, or ceftazidime (150 mg/kg via i.p. injection, q6h). (A) Survival of mice treated with GC-072, ceftazidime, or vehicle starting at 8 h postinfection (hpi). (B) Survival of mice treated with GC-072 initiated 16 hpi. A single animal in the 30-mg/kg group with treatment initiated at 16 hpi died at the beginning of the study from causes deemed unrelated to exposure or drug; therefore, this group had a total of 9 mice. *, *P* ≤ 0.05.

When treatment was initiated 8 h postchallenge, 0%, 0%, 70%, and 90% survival was observed in mice administered 1, 3, 10, and 30 mg/kg of GC-072, respectively, and 100% survival was observed in the positive-control group which received ceftazidime (Fig. 4A). When treatment with GC-072 was initiated 16 h postchallenge (Fig. 4B), 50% and 100% survival was observed in the 10 and 30 mg/kg groups, respectively. None of the placebo-treated animals survived the challenge.

The median survival times were 3.125 days for vehicle control and 3.25 and 4 days for the 1- and 3-mg/kg GC-072 groups, respectively, under the 8-h treatment initiation condition. The median survival time for the 10-mg/kg GC-072 16-h postchallenge treatment initiation group was 43 days. All of the animals in the ceftazidime group survived, as did the majority of the animals in the 8-h 10- and 30-mg/kg GC-072 and 16-h 30-mg/kg GC-072 treatment groups; thus, median survival times could not be calculated.

Under the 8-h treatment initiation condition, a statistically significant (*P* < 0.0001) dose-response trend was observed for all doses of GC-072 (see Table S3). The 3-, 10-, and 30-mg/kg GC-072 groups showed significant efficacy over that of the control (*P* = 0.0002, *P* < 0.0001, and *P* < 0.0001, respectively). Intraperitoneal treatment with ceftazidime showed a significant advantage over the groups administered 1 and 3 mg/kg GC-072 i.g. (*P* < 0.0001) but was not significantly different from the 10- and 30-mg/kg groups. Survival of mice treated with either of the GC-072 dose levels (10 and 30 mg/kg) in the 16-h treatment initiation group was superior to that of the vehicle-treated control group (*P* < 0.0001). For the 16-h initiation, administration of 30 mg/kg GC-072 showed a significant advantage over administration of 10 mg/kg GC-072 (*P* = 0.0164).

### GC-072 is efficacious against B. pseudomallei aerosol infection in a mouse model following exposure to a 339-LD_50_ bacterial challenge.

**FIG 5:**
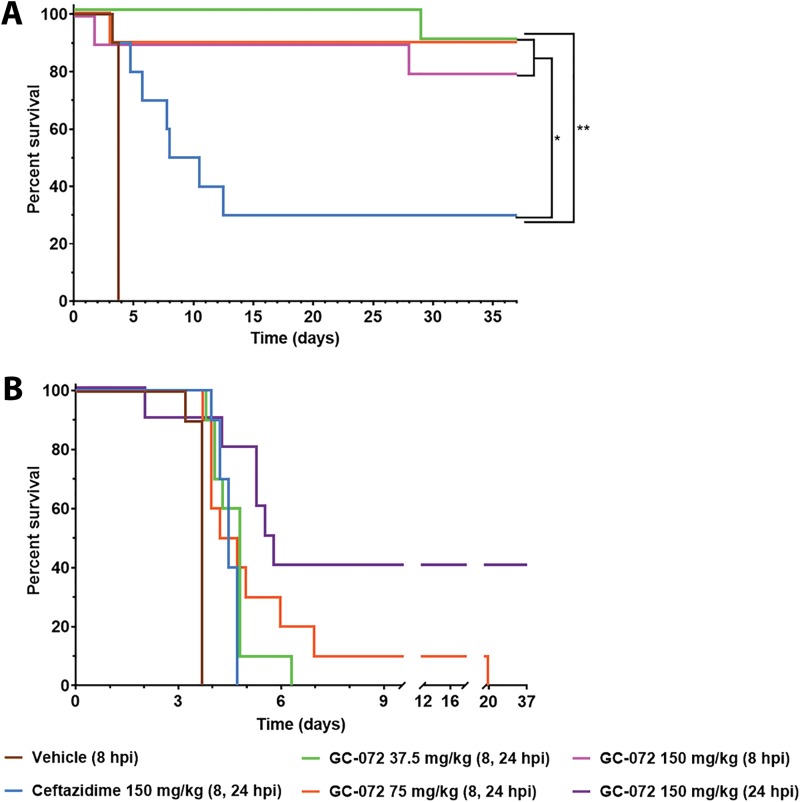
*In vivo* efficacy of GC-072 in a mouse model of melioidosis following aerosol exposure to a 339-LD_50_ bacterial challenge with B. pseudomallei strain 1026b. Treatment initiation is indicated in parentheses. Female BALB/c mice (*n* = 10/cohort; age, 6 to 8 weeks) were challenged with 339 LD_50_s of B. pseudomallei 1026b. Beginning 8 or 24 hpi, mice were treated i.g. via oral gavage q8h with either vehicle (SWFI) or GC-072 (37.5, 75, or 150 mg/kg) or q6h via i.p. injection with 150 mg/kg of ceftazidime. (A) Survival of mice treated with GC-072, ceftazidime, or vehicle initiated 8 hpi. (B) Survival of mice treated with GC-072 or ceftazidime initiated 24 hpi. *, *P* ≤ 0.05; **, *P* < 0.01.

The next study further investigated the efficacy of GC-072 against B. pseudomallei strain 1026b using a challenge level approximately 10-fold higher than the 24-LD_50_ challenge. BALB/c mice (*n* = 10/group) were treated i.g. with GC-072 (37.5, 75, or 150 mg/kg) thrice daily, 150 mg/kg i.p. of ceftazidime four times a day, or vehicle thrice daily beginning 8 or 24 h following inhalational challenge (Fig. 5A). Mice were again divided into two challenge runs with actual bacterial exposure being 1.24 × 10^6^ and 1.20 × 10^6^ CFU/mouse, representing 345 and 333 LD_50_s, respectively, with a mean of 339 LD_50_s.

When initiated 8 h postexposure, the groups treated i.g. with GC-072 had a significant survival benefit compared to both the positive comparator ceftazidime (*P* < 0.03) and vehicle control (*P* ≤ 0.0006) groups (see Table S4). Mice in the 37.5-, 75-, and 150-mg/kg GC-072 treatment groups demonstrated 90%, 90%, and 80% survival, respectively, compared to 30% in the ceftazidime and 0% in the vehicle control groups. Median survival time was 3.75 days for vehicle and 9.25 days for ceftazidime; median survival times could not be calculated for the three groups administered GC-072, as most animals survived to the end of the experiment. No dose dependence was observed in this portion of the study, as there did not appear to be a significant difference in survival for any of the GC-072 doses tested.

In the 24-h treatment initiation groups, overall survival was low; only four animals from the 150-mg/kg group survived to the end of the experiment (Fig. 5B). However, each dose of GC-072 provided a significant survival advantage compared to vehicle control (*P* ≤ 0.0006). There did not appear to be a significant difference between the 37.5- and 75-mg/kg GC-072 groups, but treatment with 150 mg/kg GC-072 provided a significant advantage over both lower-dose groups. Median survival times were 4.5 days for both the groups treated with ceftazidime and 75 mg/kg GC-072, 4.75 days for the group treated with 37.5 mg/kg GC-072, and 5.63 days for the group treated with 150 mg/kg GC-072.

## DISCUSSION

GC-072 is a novel 4-oxoquinolizine antibiotic candidate with potent activity against Gram-positive and Gram-negative bacteria, including quinolone-resistant strains. The antimicrobial data presented here and structure-based modeling with GC-072 and target topoisomerases (data not shown) indicate that the position of nitrogen in the heteroaromatic structure confers different physicochemical and biological properties than those for fluoroquinolone antibiotics. Additionally, the substituent in the C-8 position of GC-072 also takes a different conformation from that of the piperidine ring in ciprofloxacin, potentially contributing to the differential activities seen on fluoroquinolone-resistant bacterial strains. Consistent with modeling studies, results from *in vitro* topoisomerase assays indicate GC-072 more actively inhibits S. aureus topoisomerases than ciprofloxacin, which aligns with higher MICs for ciprofloxacin on Gram-positive bacteria that were observed in susceptibility studies (data not shown). Additionally, results from topoisomerase studies indicate that GC-072 and ciprofloxacin similarly inhibited quinolone-susceptible E. coli DNA gyrase and Topo IV; however, GC-072 was more active against quinolone-resistant E. coli gyrase than ciprofloxacin, consistent with a different binding mode for these molecules as well as a potentially different binding site in the QRDR. These data are consistent with GC-072 having improved activity and a different binding mode compared to that of fluoroquinolones.

*In vitro* studies show GC-072 is highly effective against B. pseudomallei and other biothreat pathogens that are resistant to current standard-of-care antibiotics. GC-072 outperformed the comparator antibiotics when tested against all five sets of biothreat agents in category A (B. anthracis, F. tularensis, and Y. pestis) and category B (B. pseudomallei and B. mallei), which included strains with known resistance. B. pseudomallei is considered naturally refractory to treatment with ciprofloxacin and other fluoroquinolones. Of the strains evaluated in this study, 20% had MICs of 8 to 16 μg/ml for ciprofloxacin. Based on the activity observed for GC-072, only 7% of strains had MICs higher than 0.5 μg/ml for GC-072, and only a single strain, resistant to ciprofloxacin, had an MIC of 1 μg/ml. The data also suggest that GC-072 is less affected by other resistance mechanisms, as a number of ceftazidime-, carbapenem-, and tetracycline-resistant strains were also included in this study.

In mutational frequency studies, when B. pseudomallei strain 1026b was exposed to 3× the MIC of GC-072, mutations arose once every 7.1 log to 8.4 log CFU compared to once every 6.6 log to 8.0 log CFU when exposed to 3× the MIC of ceftazidime. The mutation frequencies for GC-072 were consistently lower than for ceftazidime in all 5 experiments run in parallel, with ceftazidime having a 4.4-fold higher frequency of mutations than GC-072. The wild-type MICs for ceftazidime were on average 7.2-fold higher than the MICs for GC-072, and the MICs of isolates with reduced susceptibilities were on average 7.5-fold higher for ceftazidime than for GC-072. For both antimicrobials, the frequency of mutation studies demonstrated emergence of subpopulations with reduced susceptibilities to GC-072 and ceftazidime.

B. pseudomallei is known to have a number of efflux and other resistance mechanisms, which render the organism naturally resistant to a number of classes of antibiotics. However, the low MIC range observed for GC-072 in our studies indicates this candidate may be unaffected by these specific mechanisms. This observation was further investigated in a study of GC-072’s efflux propensity and resistance to strains with defined mutations. The results confirm that GC-072 has *in vitro* activity against wild-type, multidrug-resistant, and single-drug-resistant B. pseudomallei strains and that this activity is superior to that of ciprofloxacin. Previous studies showed that BpeEF-OprC-mediated efflux is the main fluoroquinolone resistance mechanism in B. pseudomallei (48). Using a panel of isogenic efflux-proficient and -compromised B. pseudomallei strains, we observed that GC-072 was less prone to efflux than the fluoroquinolone ciprofloxacin and doxycycline. Although GC-072 is a substrate of the BpeEF-OprC pump (which confers resistance to ciprofloxacin and doxycycline), efflux of GC-072 does not appear to confer significant resistance (MIC, 0.25 to 1 μg/ml) and is likely well within the therapeutic range, even for an overexpressing strain.

Intragastric treatment with a research and development formulation of GC-072 demonstrated equivalent or improved efficacy over ceftazidime in an *in vivo* murine inhalational melioidosis model, indicating this compound may be a promising candidate for the oral treatment of acute melioidosis and other biodefense indications. In the 24-LD_50_ study, the 30-mg/kg dose of GC-072 given i.g., initiated at both 8 and 16 h postexposure, showed equivalence to 8-h initiation of treatment with the positive control ceftazidime given i.p. In the context of exposure to a high-concentration bacterial challenge (∼339 LD_50_s) with B. pseudomallei, GC-072 demonstrated superiority over the positive-control ceftazidime in the 8-h treatment initiation model, even at the lowest dose of 37.5 mg/kg (*P* < 0.004). When treatment was initiated at 8 h postchallenge, only 40% of the ceftazidime group survived, while groups treated with GC-072 had an overall survival rate of 87%, indicating treatment with GC-072 provided a significant advantage. Although very few animals survived in the 24-h treatment initiation groups, there was a clear extension in survival time in the groups treated with GC-072, particularly in the 150-mg/kg group. In other studies using this animal model, a positive bacterial burden has been observed with lower bacterial counts, even when treatment was continued for an additional 7 days (62). The results from both efficacy studies indicate that treatment with GC-072 requires further investigation.

However, the oxoquinolizine GC-072 shows promising results as an oral treatment for melioidosis, which would be advantageous in resource-constrained environments and necessary in the context of a widespread, intentional biothreat event or accidental exposure to B. pseudomallei. GC-072 was shown to have improved *in vitro* potency against fluoroquinolone-resistant B. pseudomallei strains. Isogenic B. pseudomallei strains expressing defined efflux pumps, a common mechanism of fluoroquinolone resistance, were also more susceptible to GC-072 than fluoroquinolones (Table 5) (48). These factors greatly improve the potential use of GC-072 as a first-line antibiotic treatment for melioidosis.

Future studies with this compound should aim to define the therapeutic window, further optimize the treatment regimen, define pharmacokinetic/pharmacodynamic relationships (unbound therapeutic exposure levels) for human treatment, and examine extended lengths of treatment in an effort to clear residual bacteria that may cause recurrence of disease through regrowth of the pathogen. Overall, even with an efficacy equivalent to that of current standard of care, oral treatment with GC-072 could provide an advantage, as there are currently no approved oral treatment options for acute-phase melioidosis.

## MATERIALS AND METHODS

### Antimicrobial compounds.

GC-072 potassium salt was manufactured by AMRI (Albany, NY) and supplied by Emergent BioSolutions Inc. (Gaithersburg, MD). Material was supplied as powder and stored at 4°C until use. Comparator antibiotics, including ciprofloxacin, ceftazidime, meropenem, and doxycycline were purchased from U.S. Pharmacopoeia (Rockville, MD). Finafloxacin was purchased from MedChem Express (Monmouth Junction, NJ). All stocks were stored at −70°C until use.

### Bacterial strains.

A geographically and genetically diverse set of 100 strains of B. pseudomallei and 30 strains each of B. anthracis, Y. pestis, F. tularensis, and B. mallei were used for MIC determinations. Use of these sets establishes reasonable MIC_90_ and MIC_50_ values for each compound against the holistic diversity set. A compound’s performance against naturally occurring or unknown strains of each pathogen is expected to fall within these MIC_90_ and MIC_50_ values, because the diversity set provides a good representation of isolates that may be encountered anywhere in the world and reflects many common resistance mechanisms against “front-line” antibiotic therapies.

All procedures involving select agents were performed in select agent approved biosafety level 3 (BSL3) facilities at the University of Florida or USAMRIID using approved select agent compliant procedures and protocols. B. pseudomallei 1026b, obtained through the NIH Biodefense and Emerging Infections Research Resources Repository (NIAID, NIH), was used for the mutation frequency, time-kill, efflux propensity, activity against drug-resistant strains, invasion, and *in vivo* efficacy studies (45, 63). For assessing single drug class resistance, B. pseudomallei strain Bp82 (64) and its *penA* (37, 65) and *folA* (47) mutant derivatives were used. Strain Bp82 is an attenuated derivative of strain 1026b and excluded from select agent regulations (15). All experiments with strain Bp82 and its derivatives were conducted at BSL2 facilities with institutional biosafety committee approval at the University of Florida. Growth media used for Bp82 and its derivatives were supplemented with 40 μg/ml (broth) or 80 μg/ml (agar) adenine.

### Topoisomerase assays.

Gel-based topoisomerase assays were developed based on the TopoGEN Inc. and Inspiralis assay systems, where purified bacterial Topo IV and DNA gyrase from E. coli and S. aureus and human Topo I and II were exposed to different concentrations of the compounds in the presence of DNA. These assays were performed using methods previously described by Nitiss et al. (66).

### *In vitro* susceptibility testing.

Bacterial inocula were prepared by suspending in cation-adjusted Mueller-Hinton broth (CAMHB) colonies from 18- to 24-h B. anthracis, B. pseudomallei, and B. mallei plates or 42- to 48-h F. tularensis and Y. pestis plates that were incubated at 35°C. Sheep blood agar plates were used for Y. pestis and B. anthracis, and chocolate agar plates were used for F. tularensis, B. pseudomallei, and B. mallei. Suspended cultures were diluted with CAMHB to a bacterial cell density of 10^5^ CFU/ml adjusted based on comparison to a 0.5 McFarland standard. MICs were determined by the microdilution method in 96-well plates according to CLSI (67). Antibiotics were serially diluted 2-fold in 50 μl of CAMHB. For all steps with F. tularensis, CAMHB was supplemented with 2% IsoVitaleX (Becton, Dickinson).

B. pseudomallei, B. anthracis, and B. mallei plates were incubated at 35°C for 18 to 24 h and F. tularensis, and Y. pestis were incubated for 42 to 48 h. Antibiotic concentrations tested for the diverse set of B. pseudomallei ranged from 128 to 0.004 μg/ml for GC-072, ciprofloxacin, ceftazidime, and doxycycline, 16 to 0.008 μg/ml for finafloxacin, and 63 to 0.03 μg/ml for meropenem. Tested concentration ranges for the diversity sets of B. anthracis, F. tularensis, and Y. pestis were 1 to 0.0005 μg/ml and were 8 to 0.004 μg/ml for the diversity sets of B. mallei. Quality control of antibiotic stocks was established using the recommended CLSI reference strains E. coli ATCC 25922, S. aureus ATCC 29213, and Pseudomonas aeruginosa ATCC 27853.

### Mutation frequency.

Microdilution broth and agar dilution MICs and mutation frequencies were determined for GC-072 and ceftazidime for B. pseudomallei 1026b. For this study, a 1-g/liter solution of ceftazidime was made by dissolving the drug powder in sterile water and passed through a 0.22-μm syringe. Similarly, GC-072 was solubilized in dimethyl sulfoxide (DMSO) and diluted in sterile water before 0.22-μm filtration.

B. pseudomallei 1026b was grown overnight from a frozen stock on a blood agar plate (BD Diagnostics, Sparks, MD) that was incubated at 35°C in ambient air. The following morning, colonies were taken from the culture and inoculated into BBL cation-adjusted Mueller-Hinton II broth (BD Diagnostics, Sparks, MD). The suspension was gently vortexed, and the density of the bacterial suspension was visually adjusted to a 0.5 McFarland standard and then diluted with medium to a concentration of approximately 10^6^ CFU/ml. Suspensions of P. aeruginosa ATCC 27853, used as an internal control for susceptibility studies with ceftazidime, were prepared using the same protocol. A 1-ml volume of the suspension was inoculated on each of five Difco Mueller-Hinton agar plates (BD Diagnostics, Sparks, MD) supplemented with GC-072-K or ceftazidime at a concentration of 3× the agar MIC value observed with B. pseudomallei for the respective antibiotic agent. After the agar plates were incubated at 35°C for 48 h, the colonies on the drug-free and drug-supplemented agar plates were counted.

MICs were determined for a subset of colonies that grew on the GC-072- and ceftazidime-supplemented agar plates. The mutation frequency was calculated by dividing the number of colonies per milliliter on the antibiotic-supplemented agar by the number of colonies per milliliter on the drug-free agar plates.

### Time-kill study.

Thirty milliliters of 10^6^ CFU/ml of a B. pseudomallei 1026b suspension was added to each of 6 flasks. GC-072-K was added to the flasks to achieve final concentrations of 0, 1, 2, 4, 8, and 16 times the MIC (0.125 μg/ml) for B. pseudomallei 1026b. The flasks were placed on a shaker (200 rpm) in an ambient air incubator set at 35°C. At 0, 2, 5, 7.5, and 24 h of incubation, 1 ml of the suspension was collected from each flask, washed, quantitatively cultured on blood agar plates (BD Diagnostics, Sparks, MD), and enumerated after 48 h of incubation. Bacterial concentrations were log transformed and graphed.

### Efflux propensity and activity against drug-resistant strains.

For these studies, working stocks of GC-072 solubilized in DMSO (Fisher BioReagent, Pittsburgh, PA) were made by further diluting in Mueller-Hinton broth (MHB), and doxycycline and ciprofloxacin obtained from Gold Biotechnology (St. Louis, MO) were used as control antibiotics. The antimicrobial susceptibilities of strains to GC-072 were assessed by determining the MIC according to CLSI guidelines (67, 68). A colony of the tester bacterium grown at 37°C on Lennox LB (MO BIO Laboratories, Carlsbad, CA) agar plates was selected and placed in 1 ml MHB. A sufficient volume of this suspension was added to 3 ml sterile saline (0.85% NaCl) to match a 0.5 McFarland turbidity standard. Media were supplemented with 80 μg/ml adenine for Bp82-derived strains. Expression from the *tac* promoter in Bp82-*P_ta_*_c_-*penA*^+^ was induced with 1 mM isopropyl-β-d-thiogalactopyranoside (Gold Biotechnology, St. Louis, MO). MIC data were determined in technical duplicates and biological triplicates on three separate days, and plates were read after 20 h of incubation at 37°C.

### Intracellular activity.

Intracellular growth assays were performed as previously described (69). Briefly, RAW 264.7 cells (obtained from ATCC via David Pascual, University of Florida [UFL]) were infected with B. pseudomallei 1026b at a multiplicity of infection (MOI) of 10 using an aminoglycoside protection assay. After coincubation of bacteria and cells for 1 h at 37°C, the Dulbecco’s modified Eagle medium (DMEM) was then removed and the cells were washed three times with phosphate-buffered saline (PBS). Extracellular bacteria, suspended in DMEM, were killed by the addition of 250 μg/ml amikacin (Gold Biotechnology) and 250 μg/ml kanamycin (Gold Biotechnology), and plates were incubated for 1 h at 37°C. After adding fresh medium containing amikacin, kanamycin, and 0, 0.25, or 2.5 μg/ml of GC-072, the cells were incubated at 37°C. Samples were removed at 0, 1, 5, and 24 h after initiation of treatment, washed three times with PBS, and then lysed by addition of 0.2% Triton X-100 (Amresco, Solon, OH) diluted in PBS. The lysates were 10-fold serially diluted in PBS, and three 10-μl samples of each dilution were spotted onto Lennox LB agar plates. Bacterial cells growing on the plates were enumerated after overnight incubation at 37°C.

### Animal care and use.

Female BALB/c mice 6 to 8 weeks old (average weight, 19.75 g) were obtained from the National Cancer Institute/Charles River Laboratories (Frederick, MD). Animals were acclimated for 1 week prior to the challenge and had free access to food and water (*ad libitum*) throughout the study.

Guidelines in the Guide for the Care and Use of Laboratory Animals were adhered to for all experimental procedures. Research was conducted in the BSL3 laboratory at UFL in compliance with the Animal Welfare Act and other federal statutes and regulations. The facility is fully accredited by the American Association for the Accreditation of Laboratory Animal Care.

### Aerosol infection.

B. pseudomallei 1026b was grown overnight in brain heart infusion broth. For the aerosol challenge, the overnight culture was adjusted on the basis of the optical density at 600 nm (OD_600_) to give a target-challenge aerosol dose, approximately 24 or 339 LD_50_s. To verify final starting bacterial concentrations, the adjusted bacterial cultures were serially diluted and plated on tryptic soy agar (TSA) plates. Colonies were enumerated following overnight incubation of the plates at 35°C.

The target inhaled dose of B. pseudomallei was administered to mice by whole-body aerosol, which was generated using a three-jet Collison nebulizer (70). In this model, one LD_50_ was equivalent to approximately 3 × 10^3^ CFU/mouse. All aerosol procedures were controlled and monitored using the automated bioaerosol exposure system (71) operated with a whole-body rodent exposure chamber. Integrated air samples were obtained from the chamber using an all-glass impinger during each exposure. The AGI collections were serially diluted and plated on TSA, as described above. The inhaled dose (CFU/mouse) of B. pseudomallei was estimated using mouse respiratory rates per Guyton’s formula (72) and calculated according to Roy and Pitt (73).

### 24-LD_50_ bacterial challenge efficacy study.

To create a liquid solution for administration via oral gavage, the potassium salt of GC-072, which has modest aqueous solubility and good permeability, was homogenized in water containing 0.5% methylcellulose to enhance wetting of the surface of the particles. Eight groups of 10 mice each were included in this study. Beginning at 8 or 16 h after challenge, GC-072 was administered as doses of 1, 3, 10, or 30 mg/kg every 8 h (q8h) i.g. via oral gavage for 14 days. The positive comparator, ceftazidime (150 mg/kg), was administered i.p. every 6 h (q6h) for 14 days. A vehicle control group received 0.5% methylcellulose in sterile water for injection (SWFI) by oral gavage q8h. Mortality was assessed and recorded every 6 h during antibiotic administration for 14 days and at least twice a day thereafter for 45 days postchallenge. The experiment was terminated at day 45.

### 339-LD_50_ bacterial challenge efficacy study.

This experiment included 9 cohorts of 10 mice each. Beginning 8 or 24 h postchallenge, GC-072 was administered as doses of 37.5, 75, or 150 mg/kg q8h by oral gavage for 14 days. The positive comparator, ceftazidime (150 mg/kg), was administered i.p. q6h for 14 days. A vehicle control group received SWFI by oral gavage q8h. Mortality was assessed and recorded every 6 h for 14 days during antibiotic administration and at least twice daily thereafter for 37 days after the challenge.

### Statistical analysis.

All graphs were generated using GraphPad Prism version 7.00 for Windows (GraphPad Software, La Jolla, CA, USA).

MIC_90_ and MIC_50_ values from *in vitro* susceptibility testing of 100 strains of B. pseudomallei were calculated by determining the 50th percentile (median) and 90th percentile, respectively, using Prism version 7.00. MIC_90_ and MIC_50_ values from *in vitro* susceptibility testing of additional biodefense pathogens (B. anthracis, F. tularensis, Y. pestis, and B. mallei) were determined by rank-ordering MIC values from highest to lowest in a Microsoft (Redmond, WA) Excel spreadsheet and identifying the MIC_90_ (corresponding to the 27^th^ strain in the ranking of 30-strain sets) and the MIC_50_ (corresponding to the 15th strain in each rank-ordered set), respectively.

For analysis and graphing of efflux propensity, activity against drug-resistant strains, and intracellular activity studies, GraphPad Prism version 6.00 and Excel 2016 for Windows were used. The errors of the means from three biological replicates for efflux propensity and activity against drug-resistant strains were calculated using Microsoft Excel. Intracellular activity data were analyzed in Prism version 7.00 using a 2-way analysis of variance and Tukey’s test for individual comparisons.

Analysis of survival data from *in vivo* efficacy studies was performed by employing a stratified Kaplan-Meyer analysis with a log rank (Mantel-Cox) test as implemented in Prism version 7.00.

## Supplementary Material

Supplemental file 1
