# Supplementary material for: GC-072, a Novel Therapeutic Candidate for Oral Treatment of Melioidosis and Infections Caused by Select Biothreat Pathogens
Source: Antimicrob Agents Chemother. 2019 Nov 21;63(12):e00834-19. doi: 10.1128/AAC.00834-19 (PMC6879241; doi:10.1128/AAC.00834-19)
Supplement: Supplemental file 1 [file AAC.00834-19-s0001.pdf]

Shearer JK, Saylor ML, Butler CM, Treston AM, Heine HS, Chirakul S, Schewizer HP, Louie A, Drusano GL, Zumbrun SD, Warfield KL. GC-072: A Novel Therapeutic Candidate for Oral Treatment of Melioidosis and Infections Caused by Select Biothreat Pathogens.

Supplemental Material FOR Publication

Supplemental Table 1. Mean bacterial counts at collection timepoints in intracellular survival studies.

**Intracellular Survival Bacterial Counts at Collection Points (Mean bacteria per well[1])**			
**Time (h)**	**Untreated Control**	**0.25 µg/ml GC-072**	**2.5 µg/ml GC-072**
**0**	27050	27167	33550
**1**	105567	91617	21083
**5**	261667	107100	861
**24**	353833	0	0

[1]Reporting mean of 3 replicates.

Shearer JK, Saylor ML, Butler CM, Treston AM, Heine HS, Chirakul S, Schewizer HP, Louie A, Drusano GL, Zumbrun SD, Warfield KL. GC-072: A Novel Therapeutic Candidate for Oral Treatment of Melioidosis and Infections Caused by Select Biothreat Pathogens.

Supplemental Table 2. **2-Way ANOVA results from multiple comparisons of intracellular survival assay bacteria counts.**

**Multiple Comparisons of Intracellular Survival Assay Bacterial Counts**			
**Time (h)**	**Comparison between Treatment Groups**	**Mean difference (Bacteria per Well)**	**Adjusted P value[1]**
**0**	Untreated Control vs. 0.25 µg/ml GC-072	-116.7	>0.9999
	Untreated Control vs. 2.5 µg/ml GC-072	-6500	0.9537
	0.25 µg/ml GC-072 vs. 2.5 µg/ml GC-072	-6383	0.9553
**1**	Untreated Control vs. 0.25 µg/ml GC-072	13950	0.8052
	Untreated Control vs. 2.5 µg/ml GC-072	84483	0.0023
	0.25 µg/ml GC-072 vs. 2.5 µg/ml GC-072	70533	0.0107
**5**	Untreated Control vs. 0.25 µg/ml GC-072	154567	<0.0001
	Untreated Control vs. 2.5 µg/ml GC-072	260805	<0.0001
	0.25 µg/ml GC-072 vs. 2.5 µg/ml GC-072	106239	0.0002
**24**	Untreated Control vs. 0.25 µg/ml GC-072	353833	<0.0001
	Untreated Control vs. 2.5 µg/ml GC-072	353833	<0.0001
	0.25 µg/ml GC-072 vs. 2.5 µg/ml GC-072	0	>0.9999

Supplemental Table 3. Log-rank (Mantel-Cox) analysis of survival curves for 24 LD_{50} challenge with *B. pseudomallei* strain 1026b.

**8 h Treatment Initiation**		**16 h Treatment Initiation**	
**Treatments compared**	***P* value**	**Treatments Compared**	***P* value**
Vehicle vs. Ceftazidime	<0.0001	Vehicle[†] vs. 10 mg/kg GC-072	<0.0001
Vehicle vs. 1 mg/kg GC-072	0.9751	Vehicle[†] vs. 30 mg/kg GC-072	<0.0001
Vehicle vs. 3 mg/kg GC-072	0.0002	10 vs. 30 mg/kg GC-072	0.0164
Vehicle vs. 10 mg/kg GC-072	<0.0001	Ceftazidime[†] vs. 10 mg/kg GC-072	0.0115
Vehicle vs. 30 mg/kg GC-072	<0.0001	Ceftazidime[†] vs. 30 mg/kg GC-072	>0.9999
Ceftazidime vs. 1 mg/kg GC-072	<0.0001		
Ceftazidime vs. 3 mg/kg GC-072	<0.0001		
Ceftazidime vs. 10 mg/kg GC-072	0.0671		
Ceftazidime vs. 30 mg/kg GC-072	0.3173		
1 vs. 3 mg/kg GC-072	0.0003		
1 vs. 10 mg/kg GC-072	<0.0001		
1 vs. 30 mg/kg GC-072	<0.0001		
3 vs. 10 mg/kg GC-072	<0.0001		
3 vs. 30 mg/kg GC-072	<0.0001		
10 vs. 30 mg/kg GC-072	0.2907		
**Dose-response comparison**			
1, 3, 10, and 30 mg/kg GC-072	<0.0001		

[†] Indicates comparison between a GC-072-treatment group where treatment was initiated 16 hpi and the positive or negative control group (ceftazidime or vehicle), where treatment was initiated 8 hpi.

Shearer JK, Saylor ML, Butler CM, Treston AM, Heine HS, Chirakul S, Schewizer HP, Louie A, Drusano GL, Zumbrun SD, Warfield KL. GC-072: A Novel Therapeutic Candidate for Oral Treatment of Melioidosis and Infections Caused by Select Biothreat Pathogens.

Supplemental Table 4. Log-rank (Mantel-Cox) analysis of survival curves for 339 LD_{50} challenge with *B. pseudomallei* strain 1026b.

**8 h Treatment Initiation**		**24 h Treatment Initiation**	
**Treatments Compared**	***P* value**	**Treatments Compared**	***P* value**
Vehicle vs. Ceftazidime	<0.0001	Vehicle† vs. Ceftazidime	<0.0001
Vehicle vs. 37.5 mg/kg GC-072	<0.0001	Vehicle† vs. 37.5 mg/kg GC-072	<0.0001
Vehicle vs. 75 mg/kg GC-072	0.0006	Vehicle† vs. 75 mg/kg GC-072	<0.0001
Vehicle vs. 150 mg/kg GC-072	0.0006	Vehicle† vs. 150 mg/kg GC-072	0.0006
Ceftazidime vs. 37.5 mg/kg GC-072	0.0035	Ceftazidime vs. 37.5 mg/kg GC-072	0.5124
Ceftazidime vs. 75 mg/kg GC-072	0.0117	Ceftazidime vs. 75 mg/kg GC-072	0.3417
Ceftazidime vs. 150 mg/kg GC-072	0.0254	Ceftazidime vs. 150 mg/kg GC-072	0.0024
37.5 vs. 75 mg/kg GC-072	0.9703	37.5 vs. 75 mg/kg GC-072	0.4447
37.5 vs. 150 mg/kg GC-072	0.5006	37.5 vs. 150 mg/kg GC-072	0.0127
75 vs. 150 mg/kg GC-072	0.5432	75 vs. 150 mg/kg GC-072	0.0458
**Dose-response comparison**		**Dose-response comparison**	
37.5, 75, and 150 mg/kg GC-072	0.7286	37.5, 75, and 150 mg/kg GC-072	0.0243

† Indicates comparison between groups where treatment was initiated 24 hpi and vehicle control group, where treatment was initiated 8 hpi.
